# Maternal obesity increases offspring’s mammary cancer recurrence and impairs tumor immune response

**DOI:** 10.1530/ERC-20-0065

**Published:** 2020-06-22

**Authors:** Xiyuan Zhang, Fabia de Oliveira Andrade, Hansheng Zhang, Idalia Cruz, Robert Clarke, Pankaj Gaur, Vivek Verma, Leena Hilakivi-Clarke

**Affiliations:** 1 Department of Oncology, Georgetown University, Washington, District of Columbia, USA

**Keywords:** breast cancer, tamoxifen therapy, local recurrence, inflammation, tumor immune microenvironment

## Abstract

Over 50% of women at a childbearing age in the United States are overweight or obese, and this can adversely affect their offspring. We studied if maternal obesity-inducing high fat diet (HFD) not only increases offspring’s mammary cancer risk but also impairs response to antiestrogen tamoxifen. Female rat offspring of HFD and control diet-fed dams, in which estrogen receptor-positive (ER+) mammary tumors were induced with the carcinogen 7,12-dimethylbenz[a]anthracene (DMBA), exhibited similar initial responses to antiestrogen tamoxifen. However, after tamoxifen therapy was completed, almost all (91%) tumors recurred in HFD offspring, compared with only 29% in control offspring. The increase in local mammary tumor recurrence in HFD offspring was linked to an increase in the markers of immunosuppression (*Il17f*, *Tgfβ1*, VEGFR2) in the tumor microenvironment (TME). Protein and mRNA levels of the major histocompatibility complex II (MHC-II), but not MHC-I, were reduced in the recurring DMBA tumors of HFD offspring. Further, infiltration of CD8^+^ effector T cells and granzyme B+ (GZMB+) cells were lower in their recurring tumors. To determine if maternal HFD can pre-program similar changes in the TME of allografted E0771 mammary tumors in offspring of syngeneic mice, flow cytometry analysis was performed. E0771 mammary tumor growth was significantly accelerated in the HFD offspring, and a reduction in the numbers of GZMB and non-significant reduction of interferon γ (IFNγ) secreting CD8^+^ T cells in the TME was seen. Thus, consumption of a HFD during pregnancy increases susceptibility of the female rat and mouse offspring to tumor immune suppression and mammary tumor growth and recurrence.

## Introduction

During 2011–2013, half the children born in the USA had an overweight or obese mother (Ogden *et al.* 2015). The incidence of maternal obesity was particularly high (56.7%) among African American (AA) women, compared with non-Hispanic White (NHW) women (33.2%) (Flegal *et al.* 2016). Maternal obesity can have long-lasting adverse effects on the offspring that include an increased risk of type 2 diabetes, asthma, cardiovascular diseases, autism and Alzheimer’s disease (O’Reilly & Reynolds 2013, Martin *et al.* 2014, Nizari *et al.* 2016). Maternal obesity also may increase a daughter’s breast cancer risk because high birth weight is strongly linked to both maternal obesity (Yu *et al.* 2013) and an increased breast cancer risk among daughters (Michels *et al.* 1996, Silva *et al.* 2008). In a preclinical model, we earlier showed that maternal intake of an obesity-inducing high-fat diet (HFD) increased pregnancy weight gain, caused high birth weight, and increased mammary cancer risk in female offspring (de Assis *et al.* 2006). High birth weight also has been linked to increased breast cancer mortality in humans (Sovio *et al.* 2013).

An increase in breast cancer mortality reflects either primary (*de novo*) resistance to cancer therapies, or recurrence after cancer treatment is completed depicting secondary (acquired) resistance. Among patients with ER-negative disease, the risk of recurrence is greatest during the first 5 years after diagnosis and then sharply falls (Esserman *et al.* 2011). In contrast, women who develop ER+ breast cancer remain experience a steady annual risk of 3–5% after completing 5 years of endocrine therapy (Colleoni *et al.* 2016). After 20 years following initial diagnosis, 52% of those survivors who had locally advanced ER+ breast cancer have recurred and 49% died from breast cancer (Pan *et al.* 2017). For patients with early stage ER+ breast cancer, the recurrence rate is 22% and the mortality rate is 15%, 20 years after the original diagnosis (Pan *et al.* 2017).

Here, we used a preclinical model of premenopausal breast cancer to investigate whether a causative link exists between maternal obesity and a female offspring’s response to antiestrogen tamoxifen (TAM) and/or risk of local mammary cancer recurrence after TAM therapy is completed. TAM is the first-line endocrine therapy for many ER+ premenopausal patients. In the carcinogen-initiated, ER+ mammary tumor model we used at least 50% respond to TAM (Hilakivi-Clarke *et al.* 2017, Zhang *et al.* 2017); thus, tumor responses in this model mimic those seen in ER+ breast cancer patients. Further, some tumors are *de novo* resistant and never respond, and some of the responsive tumors acquire resistance to TAM and recur (Hilakivi-Clarke *et al.* 2017).

Despite years of research in trying to understand the causes of breast cancer recurrence and how to successfully treat these recurrences, advanced breast cancer remains a deadly disease and without effective treatment. We studied here a potential link between an offspring’s tumor immune microenvironment and response to TAM therapy and local recurrence. Obesity induces a low-grade chronic inflammation (Shoelson *et al.* 2007, Morris *et al.* 2011, Sun *et al.* 2011), which is causative in increasing cancer risk (Park *et al.* 2010). Increasing maternal body mass or high fat intake during pregnancy in humans is linked to chronic systemic inflammation (Leibowitz *et al.* 2012) and changes in the expression of immune genes, including T cell receptor signaling and dendritic cell maturation (Guenard *et al.* 2013) among 2–24-year-old children. When determined in the umbilical cord blood, maternal obesity caused reduced monocyte and dendritic cell responses, reduced CD4+ T helper cells, and increased levels of IFN-α and IL-6, compared with offspring of lean mothers (Wilson *et al.* 2015). Offspring of mouse dams that were fed a HFD during pregnancy have been reported to develop more severe experimentally induced bacterial infection and be more prone to develop experimentally induced autoimmunity than control offspring (Myles *et al.* 2013). Further, mice exposed to a lard-based HFD *in utero* had fewer splenic lymphocytes, thinner thymic cortex and impaired antigen-specific immune reactions as well as higher levels of TNFα (Odaka *et al.* 2010). Together, these studies indicate that maternal HFD impairs offspring’s immune responses. Effects of maternal obesity on tumor immune responses has not been studied earlier.

We found that exposure to a maternal HFD during pregnancy can program an offspring’s mammary glands to develop carcinogen-initiated mammary tumors at a younger age and to support faster growth of allografted mammary tumor cells. In addition, a maternal HFD increased the risk of local mammary cancer recurrence after TAM therapy by three-fold among offspring. These findings were related to a reduced CD4+ T cell antigen presentation, impaired infiltration of CD8+ T cells to tumor microenvironment, and lower tumor CD8+ T cell activation.

## Materials and methods

### Animals and dietary exposures

#### Rats

Three-week-old female Sprague–Dawley (SD) rats were purchased from Charles River Laboratory, and maintained on a 12-h light-dark cycle at 22°C with free access to diet and water. After 3 days of adaptation, rats were randomized into two dietary groups and fed either an AIN93G-based control diet or an obesity-inducing high-fat diet (HFD). HFD contained 215 g per 1 kg diet of Crisco (a synthetic form of lard high in saturated fat) and 50 g corn oil (contains mostly n-6 polyunsaturated fat), and the control diet contained 25 g of both fats. The customized diets were purchased from Harlan Teklad Diet Laboratories (Envigo, Madison, WI, USA); diet ingredients are listed in Supplementary Table 1 (see section on supplementary materials given at the end of this article). Female rats were kept on these diets for 12 weeks, mated, and then continued on the same diet throughout pregnancy. At birth, all dams were switched to the control AIN93G diet and 1 day later, their pups were regrouped as 10 female pups per litter.

#### Mice

Female syngeneic C57BL/6 mice were purchased from Taconic Biosciences (Rensselaer, NY, USA) and maintained as described above. After 1 week of adaptation, female mice were mated and randomized into two dietary groups and fed either an AIN93G-based control diet or a HFD. HFD used for mice contained 310 g per 1 kg diet of lard (instead of synthetic lard Crisco) and 30 g soybean oil; the control diet contained 20 g of both fats. The customized diets were purchased from Envigo Teklad Diets and the ingredients are listed in Supplementary Table 2. Female mice were kept on these diets through pregnancy until delivery. At birth, all dams were switched to the Purina 5V5 Lab Chow diet. This diet was used instead of the AIN93G diet because our pilot study indicated that over 50% of E0771 cells allografted to mice in AIN93G diet seem to have been eliminated by the immune system whilst 90% of these cells formed tumors in mice fed Purina Lab Chow.

Rat and mouse offspring were weaned at postnatal day (PND) 21. All animal procedures were approved and conducted in compliance with the Georgetown University Animal Care and Use Committee protocols.

### Mammary tumor initiation and monitoring in the female offspring: rats

On PND 50, offspring were given 10 mg of 7,12-dimethylbenz[a]anthracene (DMBA) in 1 mL peanut oil by oral gavage to initiate mammary tumorigenesis. Mammary tumors were monitored weekly by palpation, and once measurable, two dimensions of each tumor were recorded by a caliber. Tumor incidence and burden were then assessed weekly. Tumor burden was calculated by determining the total area of all tumors per rat.

#### Antiestrogen treatment and tissue collection

When a rat had at least one tumor measuring a minimum of 13 mm in its longest diameter, it was either killed for tissue collection or started on 337 ppm tamoxifen (TAM) citrate via the diet. Based on a previously published study in Sprague–Dawley rats (Hard *et al.* 1993), we estimate that the circulating TAM levels in our study in rats consuming 15 m/kg/day/337 ppm TAM were about 120–130 ng/mL. These levels are comparable to those reported in breast cancer patients (~84 ng/mL) (Kisanga *et al.* 2004). TAM-treated tumors were categorized as exhibiting a complete response when a tumor disappeared and was non-palpable for at least 7 weeks, partial response when a tumor stopped growing or shrunk, or *de novo* resistant when a tumor never responded and kept growing.

Rats with a completely responding tumor that did not grow back during the following 7 weeks were then taken off TAM. Rats were monitored on average for 9 weeks (range 4–17) after stopping TAM therapy. Tumor recurrence was recorded when a tumor grew back after completion of TAM therapy at the same location where it initially was detected and again reached a size of at least 13 mm in its longest diameter. Rats were killed when a tumor burden reached 10% of a rat’s body weight, or 30 weeks after starting TAM therapy. Blood, tumors, and tumor-free mammary glands were collected from each rat at necropsy.

#### Tumor pathological evaluation

Mammary tumors were fixed in formalin for 24–48 h before embedding in paraffin and cutting into 5 μm sections. Hematoxylin and eosin (H&E)-stained tumor sections were used for pathological evaluation by an experienced veterinary pathologist at ARUP Laboratory (Salt Lake City, UT, USA).

### Mammary tumor allografting and monitoring in the female offspring: mice

When control and HFD offspring were 8 weeks of age, they were engrafted with 2 × 10^6^ E0771 cells in 1× IMEM medium mixed with Matrigel (1:1) into the right and left 4th mammary fat pads. While E0771 cells were derived from ER+ mammary tumors from C57BL/6 female mice (Johnstone *et al.* 2015), ER expression was lost when they were established as a cell line and thus are now ER negative (Ewens *et al.* 2005, Gu *et al.* 2009, Nachat-Kappes *et al.* 2012). Consistent with being ER negative, E0771 tumors did not respond to TAM in our hands (unpublished data). Tumors were measured weekly using a caliper. Tumor volumes were calculated using the formula: ½ × (length × width^2^). Without any treatment, E0771 tumors grow rapidly, and reach a volume of >600 mm^3^ (when animals have to be killed and tumors need to be excised) in about 28 days/4 weeks from the start of the experiment (Johnstone *et al.* 2015).

### Quantitative real-time polymerase chain reaction (qRT-PCR)

Total RNA was extracted from frozen mammary adenocarcinomas using TRIzol (Life Technologies). cDNA was generated by RT using High-Capacity cDNA RT kit (Applied Biosystems) in a PTC-100 thermal cycler (Bio-Rad). To measure expression of inflammatory and immune genes, qRT-PCR was conducted using Absolute QPCR SYBR Green ROX Mix (Thermo Scientific) in an ABI Prism 7900 Sequence Detection System (Life Technologies). The primers used are listed in Supplementary Table 3. RNA expression data were quantified according to the relative method using a cDNA standard curve and normalized to RNA levels of *Hprt1* for rats and *Tbp* for mice.

### Protein isolation and Western blotting

Protein samples were prepared from frozen mammary glands or tumors following lysis in ice-cold RIPA buffer with cOmplete Mini Protease Inhibitor (Roche). Thirty micrograms of the protein was separated on a NuPage 4–12% Bis-Tris gel (Life Technologies) and transferred to nitrocellulose via an iBlot Transfer Stack and Blotting System (Life Technologies). Western blotting was performed with antibodies (diluted in 0.1% TBST at 1:1000 ratio) against the following: ERα (VP-E613, Vector Laboratories, Burlingame, CA, USA), PgR (ab90577, Abcam), Erb2/HER2 (AH01011, Invitrogen). The protein level was determined by the intensity of the bands using the Quantity One software (Bio-Rad).

### Immunohistochemistry and protein quantification

Immunohistochemistry (IHC) was used in the formalin-fixed and paraffin-embedded tumor sections to determine the numbers of CD8A, GZMB, MHC1 and MHCII positive cells, by using a secondary antibody and visualization system (K4065, DAKO). Antibodies used and dilutions in PBS were as follows: CD8A (1:100, ab33786, Abcam), GZMB (1:100, ab4059, Abcam), MHCI (1:100, NB120-6405, Novus Biological) and MHCII (1:50, ab23990, Abcam). Tissue staining was examined under a bright field microscope and 20–30 pictures for CD8A and GZMB were captured from each sample to represent the entire section. For MHCI and MHCII, 16–256 pictures were captured depending on the tumor size. Positive stained cells were quantified by a macro function in ImageJ and the average CD8A+ , GZMB+, MHCI+ and MHCII+ cells were calculated. Staining was considered positive when >10% of total cells were positive.

### TUNEL analysis

To evaluate the level of apoptosis in the mammary tumors, TUNEL assays were conducted using the apoptotic cell detection kit (Millipore, MA) following manufacturer’s instruction. For each sample, 20–30 unrepeated pictures were captured at 20× under a bright field microscope and the number of apoptotic cells was counted in ImageJ.

### Flow cytometry

E0771 mammary tumors were harvested at necropsy. Fresh tissues were processed to prepare a single cell suspension. Briefly, tumors were smashed against sterile nylon cell strainers (40 µm pore size), and single-cell suspensions were obtained by double filtering and then centrifuging the samples. Cells in a sample were resuspended in fetal bovine serum with 10% DMSO and stored in −80°C until all samples were collected and ready to be analyzed using FACS.

Immune cells in the mammary tumors were analyzed by multicolor flow cytometry using established markers (multiple fluorochrome-conjugated mAbs) against CD45 (56–0451-82; eBioscience), CD11b (553311, BD), CD11c (550261, BD), Ly6C (128041, Biolegend), Ly6G (551460, BD), F4/80 (123123, Biolegend), CD86 (105037, Biolegend), CD3 (562286, BD), CD4 (557956, BD), CD8 (100747, Biolegend), IFNγ (557724, BD), Granzyme B (372208, Biolegend), Foxp3 (561293, BD) and PD-1 (135206, Biolegend). Granulocytic-MDSCs were defined as CD45^+^CD3^-^CD11b^+^CD11c^-^Ly6G^+^ Ly6C^-^F4/80^-^ and M-MDSCs as CD45^+^CD3^-^CD11b^+^ CD11c^-^Ly6C^+^Ly6G^--^F4/80^-^. Intracellular Foxp3 and IFNγ staining was done with Foxp3 Fixation/Permeabilization Kit (Bioscience). The spatial arrangement of tumor infiltrated cells was determined by immunofluorescent staining of tumor tissues using appropriately conjugated antibodies against CD8, CD4, and FoxP3. Cell viability was determined prior to fixation and permeabilization using LIVE/DEAD™ Fixable Near-IR Dead Cell Stain Kit (Invitrogen).

### Data analysis

Statistically significant differences in tumor incidence and overall survival between control and HFD offspring were determined by Kaplan–Meier analysis followed by log rank test. Maternal body weight before and during pregnancy, and offspring body weight and tumor burden during and after TAM treatment, were compared between control and HFD groups by repeated-measures ANOVA. Differences in the proportions of mammary tumors responding to TAM treatment and in the rates of recurrence were assessed by chi-square analysis. Comparisons of data obtained by Western blotting (protein), immunohistochemistry (protein), and RT-qPCR (mRNA) in the mammary tumors between offspring were done by two-way ANOVA using maternal diet and TAM treatment (before, during, or after treatment) as variables.

Flow cytometry data were acquired using BD LSRFortessa and analyzed by FCS Express 6. All data were subjected to a single tailed Student’s *t* test with unequal variances to identify differences between two groups for statistical analysis.

All statistical analyses were carried out in GraphPad Prism 6.0 (CA) and differences with *P* < 0.05 were considered to be statistically significant.

## Results

### Effect of obesity-inducing high fat diet (HFD) on body weight in dams and offspring in rats

Rats fed an AIN93G based HFD (50% energy from fat) from age 3 weeks onwards were significantly heavier after 7 weeks of feeding when compared with control rats (13% energy from fat) (*P* < 0.05) (Supplementary Fig. 1A). Body weights of HFD and control diet fed rats remained significantly different throughout pregnancy (Supplementary Fig. 1B). Pregnancy was initiated when rats were 15 weeks of age.

Two days after birth, offspring and their nursing dams were switched to the control diet. HFD offspring were not different than the control offspring (2-way ANOVA: *P* = 0.14; Supplementary Fig. 2A). However, body weights of female HFD offspring became marginally significantly higher on postnatal day 21 (*P* = 0.05, Supplementary Fig. 2B), and significantly higher on postnatal day 50 (*P* = 0.017, Supplementary Fig. 2C). Body weights of the HFD and control female offspring were similar after DMBA administration and before TAM therapy started (Supplementary Fig. 2D).

### Carcinogen-initiated mammary tumors in rats

No animal model perfectly mimics human breast cancer. ER+ mammary tumors initiated by DMBA – a polycyclic aromatic hydrocarbon (PAH) similar to other PAHs identified as likely initiators of human breast cancer (White *et al.* 2016, Lee *et al.* 2019) – closely reflect ER+ luminal human breast cancers (Russo 2015). Further, DMBA-induced mammary tumors exhibit hotspot mutations in PI3KCA and PTEN similar to those prevalent in human ER+ breast cancers (Abba *et al.* 2016). This model was used when TAM was first shown to inhibit the growth of mammary tumors in vivo (Jordan 1983).

Animals in both groups developed mammary tumors within 19 weeks of DMBA administration. Consistent with our earlier study (de Assis *et al.* 2006), significantly higher numbers of rats in the HFD group (79.1%) than in the control group (57.1%) developed mammary tumors during the first half of the pre-treatment tumor monitoring period (*P* = 0.006).

### Tumor response to TAM therapy in rats

**Figure 1:**
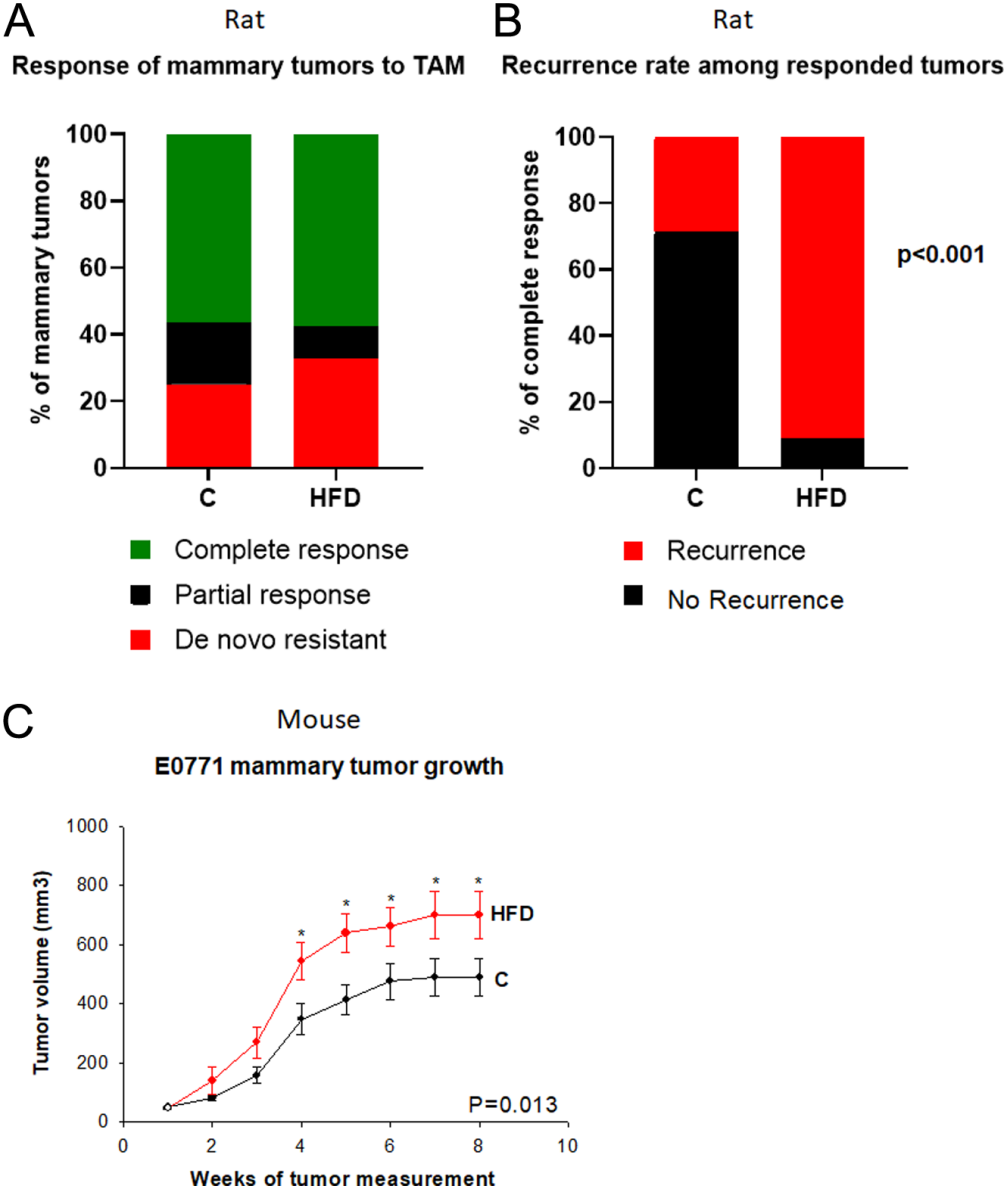
Mammary tumorigenesis in the offspring of obesity-inducing high fat diet (HFD) fed dams. (A) Percentage of complete responses (green), partial responses (black), *de novo* resistant mammary tumors (red) to tamoxifen (TAM) therapy in the offspring of HFD (*n* = 50 tumors) or control (*n* = 37 tumors) diet fed dams. (B) TAM therapy ended after a complete response was maintained for 7 weeks, and then local mammary tumor recurrences (red) were monitored. Of the 14 CR tumors in the control offspring, 4 recurred (28.6%), whilst of the 22 CR tumors in the HFD offspring, 20 recurred (90.9%) (χ^2^; *P* < 0.001). (C) Growth of E0771 mammary tumors allografted to HFD (*n* = 33 tumors) and control offspring (*n* = 52 tumors). Means ± s.e.m. of tumor volumes are shown; *P < 0.05 at specific tumor measurement time-points.

Twenty-three control and twenty-nine HFD offspring with mammary tumors were included in the study. Some offspring developed more than one tumor. The total number of mammary tumors in the controls was 32 (1.4 tumors per rat) and in HFD offspring 52 (1.8 tumors per rat). Offspring were treated with TAM when their largest tumor reached a size of ~13 mm in the longest diameter. Approximately half of the mammary tumors in the offspring of both the control (*n* = 18; 56.3%) and maternal HFD (*n* = 30; 57.7%) exhibited a complete response with TAM treatment and were no longer detectable (Fig. 1A). In the HFD offspring, the percentage of partially responding tumors that stopped growing with TAM treatment was 5 (9.6%); the partial response rate seen in the controls was 6 (18.7%). Neither the difference in partial responses, nor in the percentage of *de novo* TAM-resistant tumors (*n* = 17; 32.7% in the HFD offspring and 8 (25.0%) in the control offspring), reached statistical significance.

The average length (±s.e.m.) for TAM to induce a complete response was 3.1 ± 0.6 weeks in the control group and 2.9 ± 0.4 weeks in the HFD offspring group. After this response, TAM treatment continued for an additional 7 weeks in 12 control offspring with 14 completely responding tumors and in 14 HFD offspring with 22 completely responding tumors. The remaining animals with completely responding tumors had to be killed because they also had a resistant tumor that reached 10% of the animal’s body weight, and thus could not be followed for an additional 7 weeks. The total length of TAM treatment in offspring with a responding tumor was chosen to mimic 5 years, which is the duration of TAM treatment in most ER+ breast cancer patients. Due to differences in body size and metabolic rate, 9 weeks of rodent life corresponds to 5 years of human life (Sengupta 2013).

Recurrence was defined as a tumor that grew back in the location where it was first detected before it exhibited a complete response to TAM. During 9 weeks of post TAM follow-up, we found that 20 of 22 tumors in the HFD offspring group recurred (90.9%), whilst in the control group the rate of recurrence was 4 of 14 tumors (28.6%) (*P* < 0.001, Fig. 1B). In both groups, these tumors recurred about 6 weeks after animals were withdrawn from TAM (control group: 5.5 ± 1.2 weeks, HFD group: 5.7 ± 0.6 weeks). We did not investigate the responsiveness of the recurring tumors to TAM or other endocrine therapies.

More than 80% of mammary tumors in the offspring of both the control and HFD fed dams were malignant adenocarcinomas.

#### Allografted E0771 tumor growth in mice

If the changes in an offspring’s mammary tumor microenvironment induced by maternal obesity are sufficient to drive an increase in mammary tumorigenesis, an increase should also be seen when syngeneic offspring of obese dams are allografted with mammary tumors cells, when compared with the same cells allografted in control offspring. To study this possibility, the effects of maternal obesity were studied on the growth of allografted E0771 mammary tumor cells in syngeneic offspring. E0771 tumor cells were originally obtained from an ER+ spontaneous mammary tumor that arose in C57BL/6 mice (Johnstone *et al.* 2015). However, the cells lost ER when established as a cell line and thus are hormone receptor negative (Ewens *et al.* 2005, Gu *et al.* 2009, Nachat-Kappes *et al.* 2012).

Maternal exposure to HFD significantly increased E0771 mammary tumor burden in the offspring (*P* = 0.013, Fig. 1C).

### Cytokine expression in mammary tumors

#### Rats

**Figure 2:**
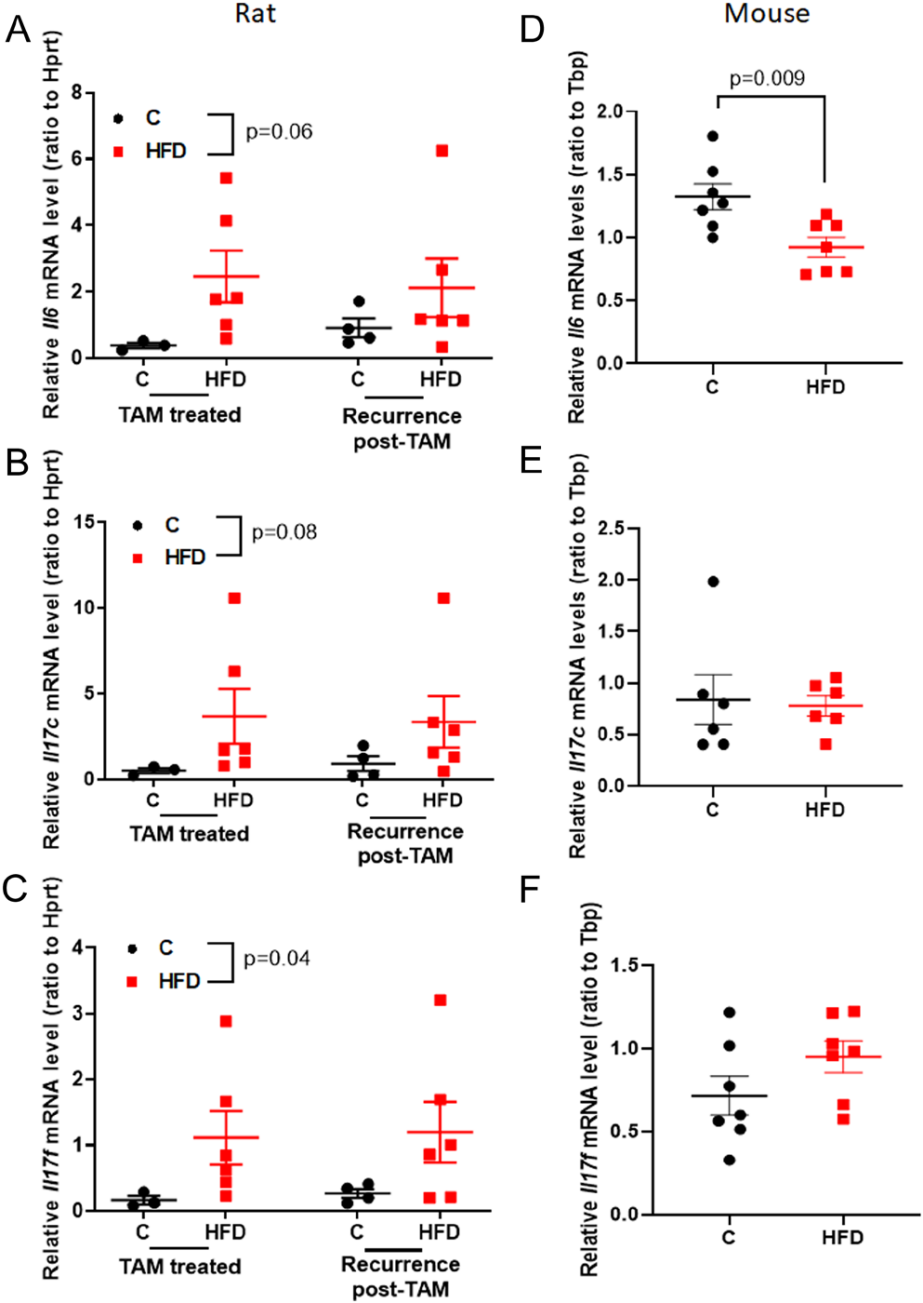
Effect of maternal obesity-inducing high-fat diet (HFD) on cytokine mRNA levels in mammary tumors in the offspring. In DMBA tumors in rats, maternal HFD marginally increased (A) IL-6 and (B) IL17c, and (C) significantly IL17f (*P* = 0.04) levels in the TAM-treated tumors of offspring (red squares), compared with tumors in control offspring [C] (black circles). Means ± s.e.m., *n* = 4–10 are shown. In E0771 tumors in mice, (D) IL-6 levels were significantly down-regulated (*P* = 0.009) in the HFD offspring and there was no change in the expression of (E) IL-17c or (F) IL17f. Control [C]: black circle, HFD: red square. Means ± s.e.m., *n* = 7 are shown.

We focused here on investigating if the increased risk of local recurrence after TAM therapy was linked to changes in the tumor immune environment. First, mRNA levels of three cytokines were measured in the mammary tumors: (i) IL-6 that induces differentiation of activated CD4+ T helper cells toward inflammatory Th17 cells, (ii) IL-17c that is secreted from Th17 cells and is expressed only in activated T cells where it promotes inflammation in the tumor microenvironment and enhances tumor growth, and (iii) IL-17f that induces inflammation and is associated with breast cancer progression, metastasis, and poor overall survival (Coffelt *et al.* 2015). Increased expression of *Il6* (*P* = 0.06) and *Il17c* (*P* = 0.08) in these tumors in the HFD offspring reached borderline significance only (Fig. 2A and B). However, compared with the control offspring, *Il17f* expression was significantly upregulated in mammary tumors of the offspring of HFD-fed dams obtained from TAM-treated tumors and recurring tumors (*P* = 0.04) (Fig. 2C).

#### Mice

None of the three cytokines was significantly upregulated in the E0771 mammary tumors of HFD offspring, compared with the control offspring (Fig. 2D, E and F). Indeed, *Il6* was significantly downregulated in E0771 tumors allografted to the offspring of dams fed a HFD diet during pregnancy (Fig. 2D). Since IL-17 cytokines were not significantly different in E0771 cells in mice which were never treated with TAM, it is likely that the difference in *Il17f* in DMBA-treated tumors between control and HFD offspring was partly reflective of TAM treatment.

### Tumor immune microenvironment: rats

#### CD8 antigen (CD8A) and Granzyme B (GZMB)

**Figure 3:**
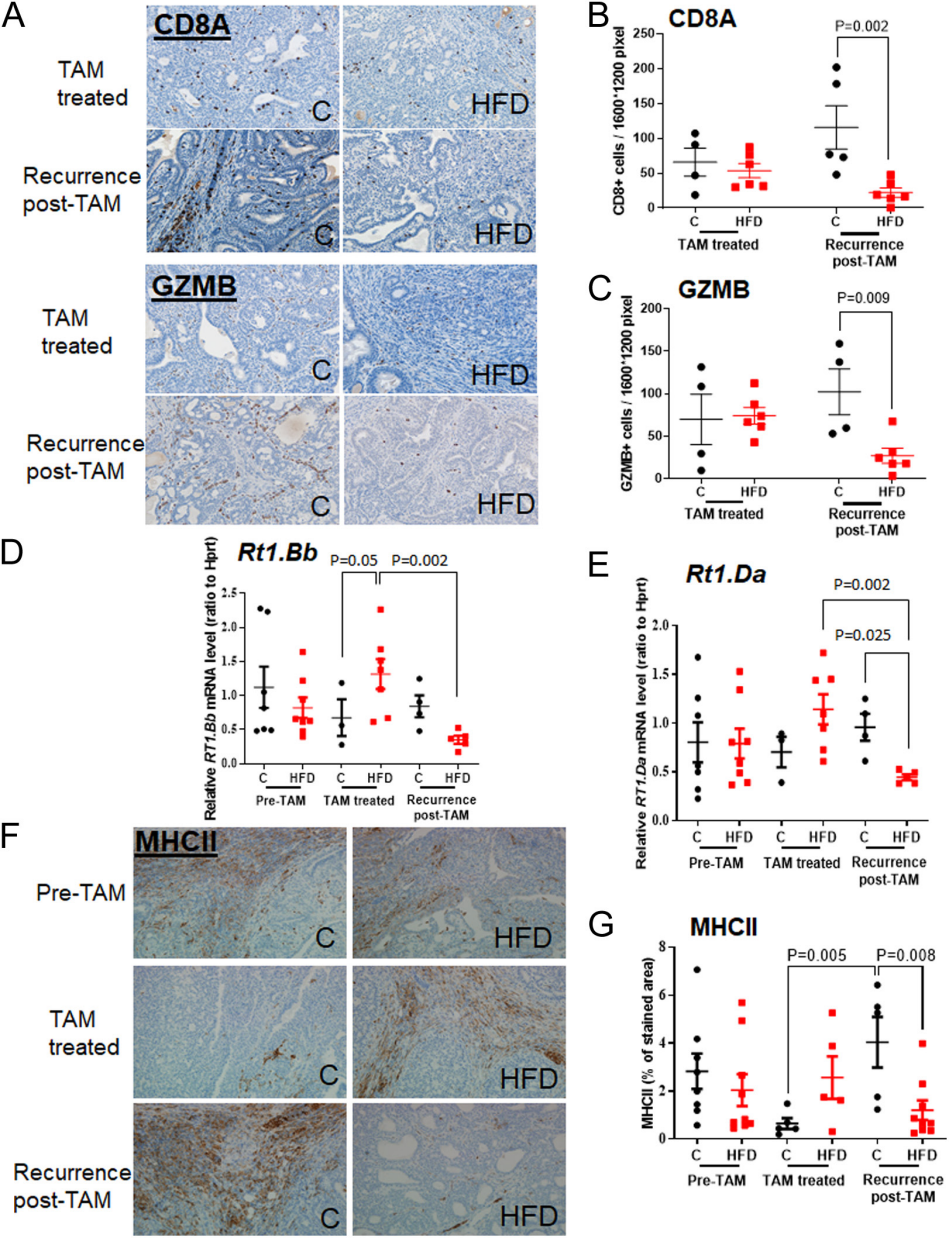
Effect of maternal obesity-inducing high-fat diet (HFD) on CD8A+ and granzyme B (GZMB) protein levels and markers of MHCII in mammary tumors in rat offspring. (A) Representative pictures of immunohistochemically stained CD8A+ and GZMB+ tumor-infiltrating lymphocytes (TILs) in TAM-treated and post-TAM recurring mammary tumors. 20×. Quantitative analysis of 10–30 areas of each slides (*n* = 4–6 for the two offspring groups) showed that maternal HFD diet significantly reduced the number of (B) CD8A+ TILs and (C) GZMB+ TILs in the recurring tumors. Means ± s.e.m., *n* = 4–10 offspring of both control and HFD groups are shown. *P < 0.05, **P < 0.01, ***P < 0.001. (D) Gene expression of RT1.Bb and (E) RT1.Da in rat mammary tumors from control (black circles) and HFD (red squares) offspring before TAM treatment, and in TAM-treated or in post-TAM recurring tumors. Means ± SEM, n = 3-8 offspring of both control and HFD groups are shown. (F) Representative pictures of immunohistochemically stained MHCII+ cells in rat mammary tumors before and during treatment and in recurring tumors from control and HFD offspring. 20×. (G) Quantitative analysis of 16–256 pictures captured from each slide depending on the tumor size (n = 5-9 for the two offspring groups) showed that maternal HFD significantly reduced the MCHII protein levels in recurring tumors compared to control offspring.

In the recurring tumors, IHC analysis showed a significant reduction in the infiltration of CD8A+ (*P* = 0.002, 2-way ANOVA for interaction: *P* = 0.039, Fig. 3A and B) and GZMB+ (*P* = 0.009, 2-way ANOVA for interaction: *P* = 0.041, Fig. 3A and C) immune cells in HFD offspring, compared with controls. No difference in either CD8A+ or GZM B levels between the HFD and control offspring was seen during TAM treatment. These results suggest that anti-tumor immune responses were reduced in recurring tumors in the offspring of obese dams.

#### Antigen presentation

To determine if the suppression of anti-tumor CD8+ T immune cells was linked to changes in antigen presentation, we assessed the expression of histocompatibility complex (MHC) class I (*RT1.A1* and *RT1.EC2*) and class II genes (*RT1.Bb* and *RT1.Da*) in the mammary tumors of the offspring. During TAM treatment, the expression of *RT1.Bb* was higher in the tumors of HFD than control offspring (*P* = 0.05) (Fig. 3D). However, after TAM therapy recurring tumors in the HFD offspring showed a significantly lower expression of *RT1.Bb* than in a different set of tumors where *RT1.Bb* was measured during TAM therapy (*P* = 0.002; 2-way ANOVA for interaction: *P* = 0.016) (Fig. 3D).

Similarly, recurring tumors from HFD offspring showed a lower mRNA expression of the other MHCII gene, *RT1.Da*, compared with TAM-treated tumors (*P* = 0.002) and recurring tumors from control offspring (*P* = 0.025, 2-way ANOVA for interaction: *P* = 0.006) (Fig. 3E). Protein levels of MHCII, determined by IHC, were lower in the recurring tumors from HFD offspring compared with control offspring (*P* = 0.008; Fig. 3F and G). Recurring tumors from control offspring had higher MCHII levels than tumors during TAM treatment (*P* = 0.005; 2-way ANOVA for interaction: *P* = 0.013, Fig. 3F and G), suggesting that TAM increases MHCII in the tumors of HFD offspring but suppresses MHCII in controls. No significant change in the expression of MHCI genes was observed (Supplementary Fig. 3). These data suggest that CD4+ T cell antigen presentation activity was reduced in the recurring tumors in HFD offspring.

#### FOXP3, VEGFR2 and Tgfβ1

**Figure 4:**
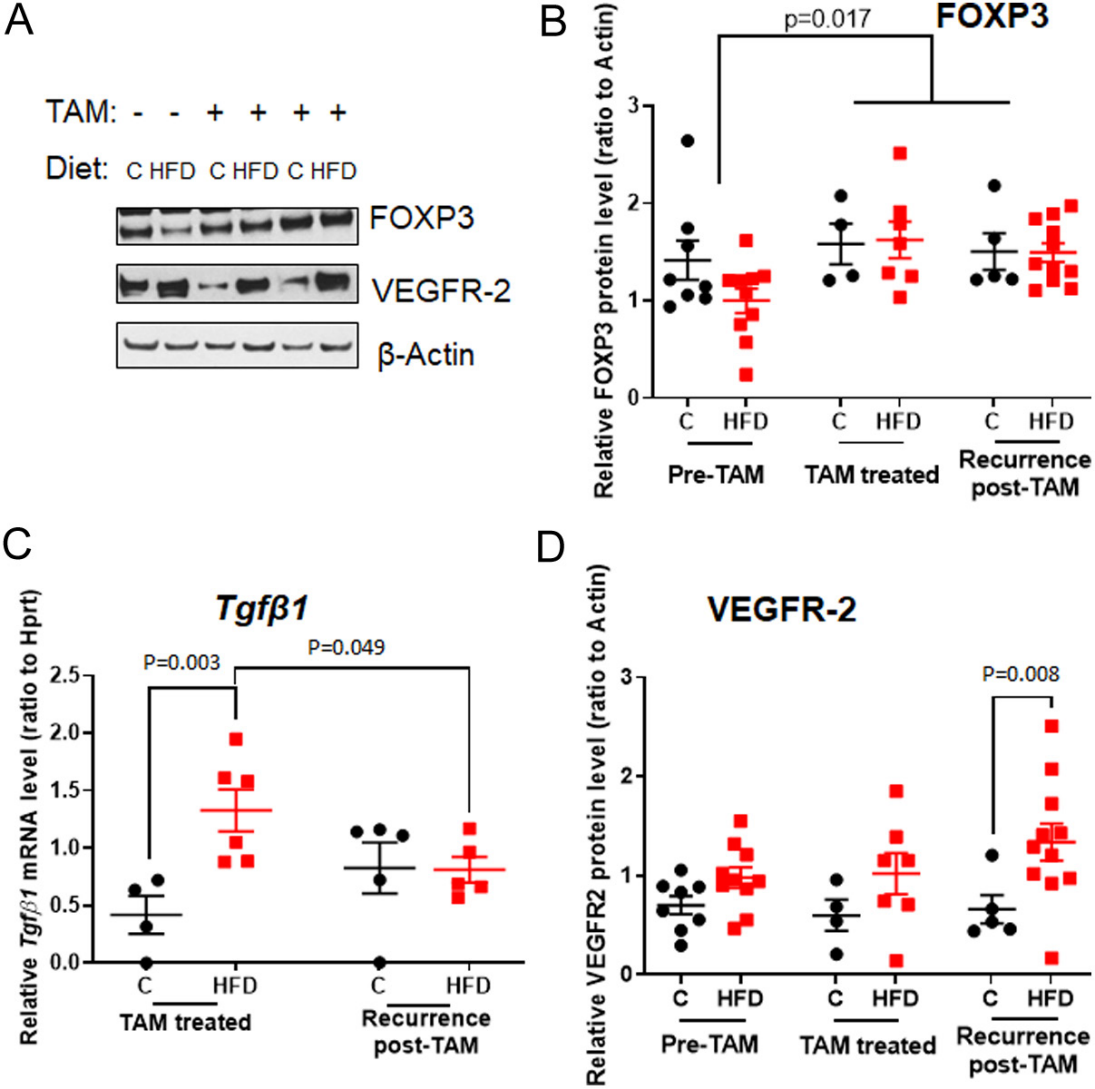
Effect of maternal obesity-inducing high fat diet (HFD) on tumor immunosuppressive markers in rat offspring. (A) Representative Western blots and quantitated protein levels of (B) FOXP3 and (D) VEGFR-2, in the mammary tumors of control [C] (black circles) and HFD (red squares) offspring before TAM treatment, and in TAM-treated or post-TAM recurring tumors. (C) *Tgfβ1* mRNA levels in TAM-treated tumors of control and HFD offspring. Mean ± s.e.m., *n* = 4–10 offspring of both control and HFD groups are shown. *P < 0.05, **P < 0.01.

No change in the T regulatory cell marker FOXP3 was seen in the HFD offspring by Western blot, compared with the control offspring (Fig. 4A and B). However, consistent with earlier findings (Joffroy *et al.* 2010), TAM upregulated FOXP3, and its expression remained upregulated in recurring tumors (2-way ANOVA: *P* = 0.017). The *Tgfβ1* mRNA was significantly higher in HFD offspring during TAM treatment than in the controls (*P* = 0.04), but this difference was no longer apparent in recurring tumors (Fig. 4C). The difference in *Tgfβ* levels between control and HFD offspring may be linked to TAM, since it was seen only during TAM treatment and TAM is known to increase TGFβ (Arteaga *et al.* 1999). VEGFR2 protein levels were significantly higher in the recurring tumors of HFD than control offspring (*P* = 0.003; 2-way ANOVA: *P* = 0.004) (Fig. 4A and D). Thus, while maternal obesity does not increase FOXP3 levels in the offspring’s mammary tumors, upregulation of *Tgfβ1* and VEGFR2 suggests that maternal HFD may promote immunosuppression in TAM-treated offspring’s mammary tumors.

### Tumor immune microenvironment: mice

**Figure 5:**
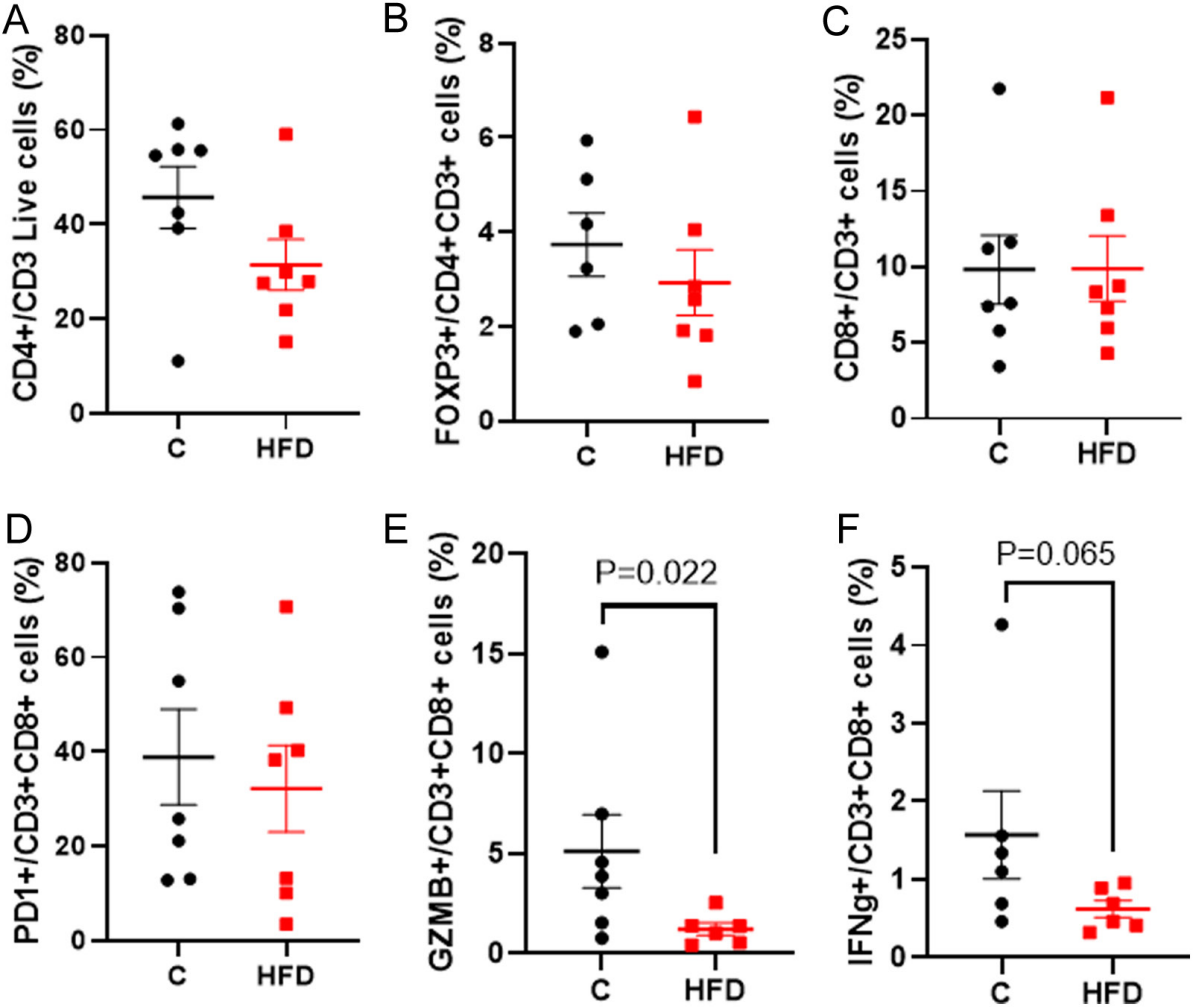
Effect of maternal obesity-inducing high fat diet (HFD) on markers of tumor immune cell infiltration of E0771 mammary tumors in mouse offspring. Frequency of (A) CD4+ T lymphocytes (CD4+CD3+), (B) T reg cells (FOXP3+CD4+CD3+), and (C) CD8+ T cells (CD8+CD3+). (D) CD8 T cells exhaustion (PD1+CD8+CD3+), and CD8 T cells activation (E) measured by GZMB (GZMB+CD8+CD3+) and (F) IFN-γ (IFN-γ+CD8+CD3+) in the mammary tumors from control [C] (black circles) and HFD (red squares) offspring. Mean ± s.e.m., *n* = 6–7 for both control and HFD groups are shown.

Maternal exposure to HFD did not significantly change E0771 tumor infiltration of CD4+ T lymphocytes or T reg cells (CD4+FOXP3+ cells) in the offspring (Fig. 5A and B). Moreover, while the frequency of CD8+ T cells (Fig. 5C) or PD1+ CD8+ T cells (CD8+PD1+) (Fig. 5D) did not change, E0771 tumors from HFD offspring showed a decreased activation of CD8+ T cells, determined by expression of GZMB (*P* = 0.022, Fig. 5E) on CD8+ T cells. Furthermore, maternal HFD tended to suppress IFN-γ (*P* = 0.065, Fig. 5F), but did not affect the frequency of monocytic-MDSC cells (Supplementary Fig. 4A) and DC cells (Supplementary Fig. 4B), or the activation status of the DC-expressing surface co-stimulatory molecule CD86 (CD86+ cells, Supplementary Fig. 4C). These results confirm the data obtained in DMBA tumors in rats, and suggest that CD8 mediated anti-tumor immune responses, and not the antigen presentation, was reduced in E0771 tumors in the HFD offspring.

### Epithelial-to-mesenchymal transition (EMT) markers

#### Rats

**Figure 6:**
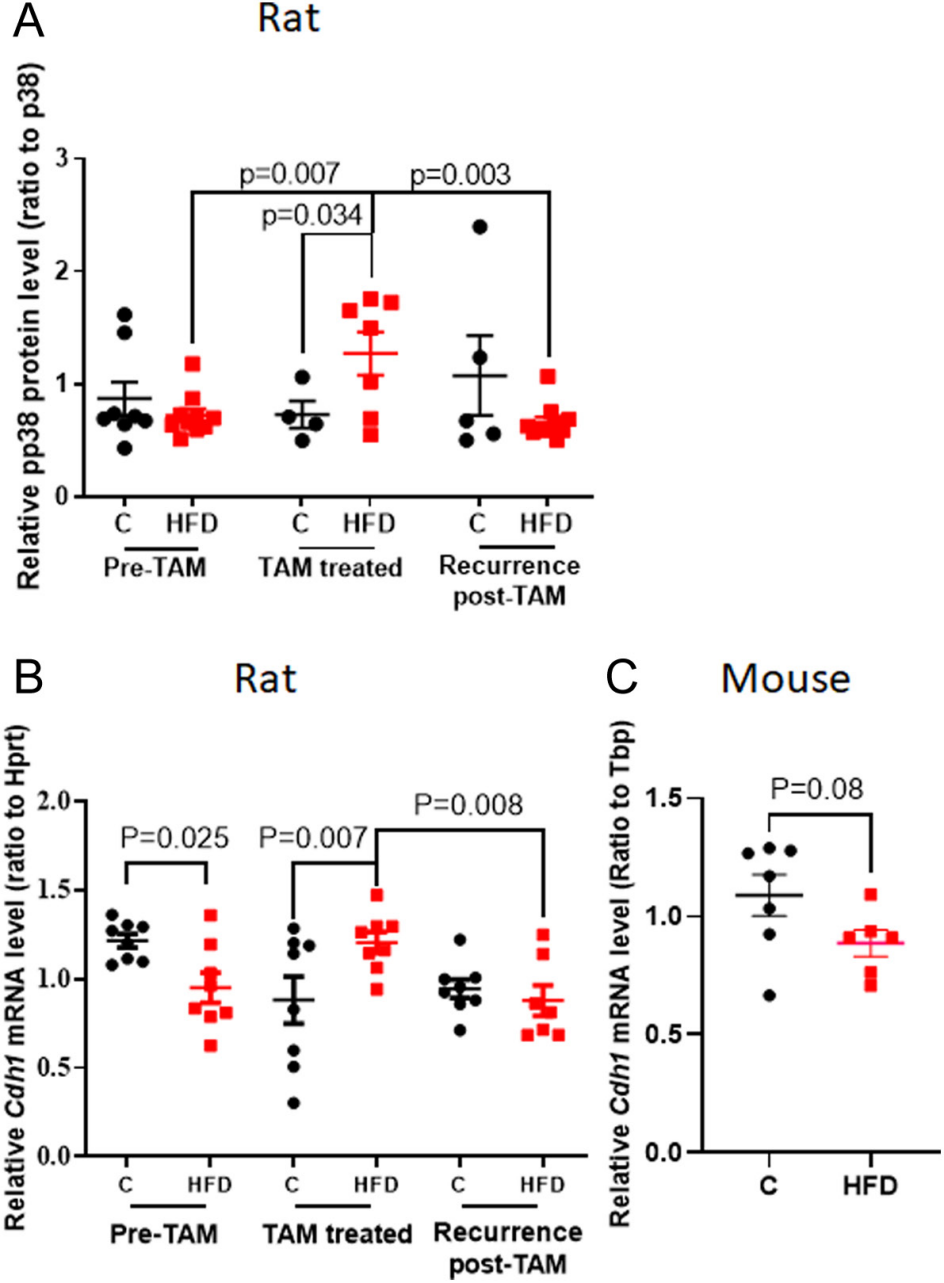
Effect of maternal obesity-inducing high fat diet (HFD) on markers of epithelial-to-mesenchymal transition (EMT) in offspring’s mammary tumors. (A) Protein levels of p38 and (B) Cdh1 gene expression in the rat mammary tumors of control [C] (black circles) and HFD (red squares) offspring before TAM treatment, and in TAM-treated or recurring tumors. Mean ± s.e.m., *n* = 4–10 offspring of both control and HFD groups are shown. (C) Cdh1 gene expression in allografts of E0771 tumors of control [C] (black circles) and HFD (red squares) mouse offspring. Mean ± s.e.m., *n* = 6–7 tumors of both control and HFD groups are shown.

Since TGFβ is a critical regulator of an EMT, we determined if p38, a target gene of TGFβ1 that is known to induce EMT, was altered. It was found to be significantly higher during TAM therapy in mammary tumors of HFD offspring than in the control offspring (*P* = 0.034; 2-way ANOVA for interaction: *P* = 0.018) (Fig. 6A). Interestingly, during an EMT, epithelial cells loose epithelial markers, such as CDH1/E-cadherin, and acquire mesenchymal markers, including N-cadherin/CHD2 and vimentin. Hence, next we determined if the expression of the key EMT gene – *Cdh1*/E-cadherin was also altered. Expression of *Cdh1*/E-cadherin was significantly downregulated before TAM treatment in the mammary tumors of HFD offspring (*P* = 0.025), compared with control offspring (Fig. 6B). TAM treatment increased *Cdh1* expression in the HFD offspring compared with control offspring (*P* = 0.007); however, in the recurring tumors *Cdh1* expression was lower in the HFD, compared with TAM-treated HFD tumors (*P* = 0.008). Mammary tumors from control offspring had higher expression of *Cdh1* before treatment, compared with TAM-treated (*P* = 0.005) and recurring tumors (*P* = 0.022; 2-way ANOVA for interaction: *P* = 0.003). No change in the expression of either *Cdh2* or vimentin was seen between the HFD and control offspring (data not shown). Hence, these results show that exposure to a maternal HFD causes an offspring’s mammary tumors to lose epithelial markers but does not increase markers of mesenchymal cells.

#### Mice

*Cdh1*/E-cadherin mRNA expression was marginally lower (*P* = 0.08) in E0771 mammary tumors from HFD offspring than in control offspring (Fig. 6C).

### Hormone receptor levels in the tumors before and after TAM therapy

No difference in ERα levels was seen between control and HFD offspring before TAM therapy. However, during TAM therapy ERα levels were higher in the offspring of HFD fed dams than of control dams (*P* = 0.017; Holm–Sidak: *P* = 0.002; Supplementary Fig. 5A). ERα protein levels also were higher in the TAM-treated mammary tumors of HFD offspring compared with pre-TAM treatment (*P* = 0.002; Holm–Sidak: *P* = 0.05). Protein levels of HER2 did not change (Supplementary Fig. 5B). The findings in the control offspring are consistent with several earlier findings indicating that TAM does not modify ERα levels, but over time causes an accumulation of ERα (Pink & Jordan 1996). Our results suggest that HFD offspring are more sensitive to the increase in ERα protein levels by TAM than control offspring.

### Apoptosis and proliferation levels in the tumors: rats only

Reduced apoptosis and increased cell proliferation could be driving the increase in recurrences among HFD offspring. Apoptotic cells were detected using the TUNEL assay. No difference in the apoptosis of tumor cells among the maternal dietary groups was seen. However, significantly fewer apoptotic cells were present in the recurring tumors than in the tumors during TAM therapy (*P* = 0.002; Holm–Sidak: *P* = 0.023 within control and *P* = 0.018 within HFD; Supplementary Fig. 5C and E). This finding may reflect TAM’s ability to increase apoptosis in mammary tumor cells. Expression of Ki67 mRNA, a marker of cell proliferation, did not change in the mammary tumors in the control and HFD offspring (Supplementary Fig. 5D).

## Discussion

While we found no difference in the responses of primary tumors to TAM treatment between the control and HFD offspring, when therapy was completed and TAM was removed, the risk of local recurrence was over 90% in the HFD offspring, compared with less than 30% in the controls. The lack of difference in the response to cancer therapy is consistent with an earlier study in MMTV-Wnt1-Tg mice in which no differences in the response to doxycycline (Dox) chemotherapy in the control and HFD offspring were noted (Montales *et al.* 2016). The MMTV-Wnt1-Tg HFD offspring exhibited an increased risk of developing mammary tumors (Montales *et al.* 2016). We also confirmed our earlier finding showing that the offspring of rat dams that were fed with a HFD during pregnancy develop DMBA-initiated mammary tumors at a younger age than the control offspring (de Assis *et al.* 2006). In addition, when offspring of HFD-fed dams received allografted E0771 cells, the mammary tumors grew faster leading to increased tumor burden in syngeneic mice exposed to HFD *in utero*.

Several changes in the tumor microenvironment in HFD offspring were seen in both the DMBA and E0771 mammary tumor models. In the DMBA model, we first measured levels of intra-tumoral cytokines, since these are reflective of the function of immune cells. We found increased mRNA expression of *Il17f*, and a tendency toward an increase of *Il17c* and *Il6*, in the mammary tumors of HFD offspring. In the E0771 tumors, *Il17f* also was increased in the HFD offspring, although the increase was not statically significant. These results are in agreement with the findings of an earlier study reporting that in the offspring of high-fat diet-fed dams, colonic cells exposed to inflammatory-response-inducing lipopolysaccharide (LPS), exhibited increased release of IL-6, IL-1β, and IL-17 (Myles *et al.* 2013). High circulating and intratumoral levels of IL-17/IL-17f promote breast cancer progression and metastasis, and are indicative of poor overall survival in breast cancer patients (Coffelt *et al.* 2015). The increase in IL-17f could have been caused by TGFβ and IL-6 promoting the generation of Th17 (Zhang 2018). In our study, tumors in the offspring of HFD dams exhibited an upregulation of *Tgfβ1* in the tumors during TAM treatment. Another immunosuppressive marker, VEGFR2 (Suzuki *et al.* 2010), was upregulated in recurring tumors. However, neither FOXP3 mRNA expression nor Foxp3 positive cells in flow cytometry were different in the allografted E0771 tumors between the control and HFD offspring.

In addition to being closely involved in activating immunosuppressive mechanisms, TGFβ is a key inducer of the epithelial-to-mesenchymal transition (EMT) (Katsuno *et al.* 2013). Phosphorylation of p38 that mediates the effects of TGFβ in inducing EMT (Bakin *et al.* 2002, Heldin & Moustakas 2016) was increased in the mammary tumors of the HFD offspring during TAM therapy. Further, E-Cadherin/Cdh1 prevents epithelial cells from acquiring a more mesenchymal phenotype and was significantly downregulated in the DMBA tumors; a similar trend was seen in the E0771 tumors. The ability of the tumor microenvironment to reprogram tumor cells is well established and involves epigenetic pathways activated by cancer-associated fibroblasts (CAFs) (Mathot *et al.* 2017). Our future studies will include examining the possible that maternal HFD pre-programmed offspring’s mammary gland in a manner that made it more susceptible for growth of allogafted E0771 mammary tumor cells, and recurrence of DMBA-initiated mammary tumors after TAM therapy.

Infiltration of CD8+ T cells into the tumor microenvironment is predictive of a good prognosis in many cancers, including in ER negative breast cancer (Ali *et al.* 2014, Mao *et al.* 2014). However, in ER+ breast cancers high CD8+ T cell levels before any treatment are either not predictive of cancer outcome (Dushyanthen *et al.* 2015) or linked to an unfavorable outcome (Sobral-Leite *et al.* 2019). In our study, CD8A protein levels were increased in non-treated ER+ DMBA mammary tumors in the HFD offspring. After TAM therapy, expression and infiltration of total CD8+ and GZMB+ CD8+ T cells were significantly reduced in the recurring tumors of the offspring of dams that were fed HFD during pregnancy, compared with the tumors during TAM therapy or recurring tumors in the control offspring. This observation may have reflected a reduction in antigen presentation for CD4+ T cells. We found that mRNA expression of MHCII was suppressed in the recurring tumors of HFD offspring, compared with the expression of these genes in the tumors of either control offspring or in tumors during TAM treatment in the HFD offspring. Similar data were obtained when we assessed MHCII protein levels by IHC. In E0771 tumors in the HFD offspring, activation of CD8+ T cells was reduced as indicated by a reduction in GZMB and IFNγ in CD8+ T cells. It remains to be determined if the suppression of antigen presentation in CD4+ T cells and CD8+ T cell activation in the tumor microenvironment of HFD offspring is causally linked to their increased mammary cancer growth and risk of recurrence.

There are both similarities and differences in the results generated using the two different mammary cancer models. For the pourposes of this study, the most critical difference is that in the DMBA model the tumors arise spontaneously from normal mammary epithelial cells that are transformed by the carcinogen, whereas E0771 mammary tumors originated in a different animal and were introduced into the normal mammary fat pad. The tumor microenvironment (TME) in the DMBA model is likely to be more similar to changes in breast cancers in the daughters of obese mothers. Since studies involving allografted tumor cells offer more possibilities to investigate the causality between the increased mammary tumorigenesis and changes in the offspring’s TME, as programmed by maternal obesity, it is important to establish that maternal obesity increases an offspring’s mammary tumorigenesis, regardless of the model used.

The incidence of obesity during pregnancy has increased by 5-fold during the past 50 years. In light of our results presented in this study, it would be critical to identify effective strategies to prevent recurrence in ER+ breast cancers in the daughters of obese mothers. We discovered that maternal HFD dramatically increased an offspring’s mammary cancer recurrence in the carcinogen model after a completion of TAM treatment. The increase in local recurrence was linked to a suppression of markers of CD4+ T cell antigen presentation and CD8+ T cell infiltration in the recurring tumors, indicative of reduced anti-tumor immunity. In the immune microenvironment of E0771 allografted tumors, activation of CD8+ T cells was reduced in HFD mouse offspring. Further, both carcinogen-initiated and allografted tumor cells expressed reduced levels of *Cdh1*, indicative of an increased tendency for an EMT. An interaction between the tumor immune microenvironment and EMT has been established previously (Terry *et al.* 2017, Singh & Chakrabarti 2019, Soundararajan *et al.* 2019), although it remains to be clarified if EMT alters tumor immune microenvironment, or vice versa, or if the interaction is bidirectional. Since allografted tumor cells were never directly exposed to a maternal HFD, any changes in E0771 tumor cells must have occurred in response to changes in the host caused by maternal HFD exposure.

## Supplementary Material

Supplementary Figure 1. Weight gain in obesity-inducing high fat diet (HFD) fed dams before and during pregnancy. (A) Weight gain of dam rats before pregnancy. Seven weeks of HFD feeding led to a significantly higher weight gain, compared with control [C] diet. Rats were kept on these diets for three additional weeks before mating. (B) Weight gain during pregnancy in control and HFD fed dams. Mean ± SEM, C: black circle, HFD: red square, *p<0.05, **p<0.01.

Supplementary Figure 2. Body weight of offspring of dams fed obesity-inducing high fat diet (HFD) or control [C] diet. (A) Birthweight of male and female offspring of control and HFD fed dams. (B) Body weight of female offspring at postnatal day (PND) 21 and (C) PND 50. (D) Body weight of female offspring six weeks after the DMBA administration. Mean ± SEM, control: black circle, HFD: red square, *p<0.05.

Supplementary Figure 3. Effect of maternal obesity-inducing high fat diet (HFD) on MHCI in the rat mammary tumors of offspring. (A) Gene expression of RT1.A1 and (B) RT1.EC2 in rat mammary tumors from control [C] (black circles) and HFD (red squares) offspring before TAM treatment, and in TAM-treated or post-TAM recurring tumors. Means ± SEM, n=3-8 offspring of both control and HFD groups are shown. (C) Representative pictures of immunohistochemically stained MHCI+ cells in rat mammary tumors before and during treatment and in recurring tumors from control and HFD offspring. 20X. (D) Quantitative analysis of 29-162 pictures captured from each slide depending on the tumor size (n=5-9 for the two offspring groups).

Supplementary Figure 4. Effect of maternal obesity-inducing high fat diet (HFD) on immune markers in E0771 mammary tumors of mouse offspring. Frequency of (A) monocytic-Myeloid-derived suppressor cells (M-MDSC: CD45+CD3-CD11b+CD11c-Ly6C+Ly6G--F4/80-) (B) Dendritic cells (DC: CD45+CD3-CD11b+CD11c+F4/80-) and (C) DC cells activation measured by CD86+ cells in the mammary tumors from control [C] (black circles) and HFD (red squares) offspring. Mean ± SEM, n=7 for both control and HFD groups are shown.

Supplementary Figure 5. Effect of maternal obesity-inducing high fat diet (HFD) on hormone receptor levels, apoptosis and cell proliferation in the mammary tumors of rat offspring. (A) Maternal HFD increased ERα protein level in the TAM-treated tumors, when compared with TAM-treated tumors in controls [C] (black circle) or HFD (red square) tumors before TAM therapy. (B) HER2 protein level did not differ between C and HFD offspring. (C) Quantitative analysis and (E) representative pictures of Tunel assay in rat mammary tumors before and during treatment and in recurring tumors from control and HFD offspring. (D) Gene expression of Ki67 in rat mammary tumors from control and HFD offspring before TAM treatment, and in TAM-treated or post-TAM recurring tumors. Mean ± SEM: *p<0.05, **p<0.01.

Supplementary Table 1. Ingredients of control and high fat diets for rats

Supplementary Table 2. Ingredients of control and high fat diets for mice

Supplementary Table 3. Primers used in quantitative real-time PCR

## Declaration of interest

The authors declare that there is no conflict of interest that could be perceived as prejudicing the impartiality of the research reported.

## Funding

This work was supported by U54-CA149147 and U01-CA184902 to R Clarke, and R01-CA164384 and AICR grant to L Hilakivi-Clarke, and P30-CA51008 to Lombardi Comprehensive Cancer Center (funding for Shared Resources).

## Author contribution statement

*Conception and design:* L Hilakivi-Clarke, X Zhang, F de Oliveira Andrade, R Clarke. *Development of methodology:* X Zhang, F de Oliveira Andrade, L Hilakivi-Clarke, R Clarke, V Verma. *Acquisition of data:* X Zhang, F de Oliveira Andrade, I Cruz, H Zhang, P Gaur. *Analysis and interpretation of data:* X Zhang, F de Oliveira Andrade, V Verma, L Hilakivi-Clarke, R Clarke. *Writing the manuscript:* X Zhang, L Hilakivi-Clarke, F de Oliveira Andrade, R Clarke. *Administrative, technical, or material support:* I Cruz, R Clarke. *Study supervision:* L Hilakivi-Clarke.

## References

[bib1] Abba MC Zhong Y Lee J Kil H Lu Y Takata Y Simper MS Gaddis S Shen J Aldaz CM 2016 DMBA induced mouse mammary tumors display high incidence of activating Pik3caH1047 and loss of function Pten mutations. Oncotarget 7 64289–64299. (10.18632/oncotarget.11733) 27588403 PMC5325442

[bib2] Ali HR Provenzano E Dawson SJ Blows FM Liu B Shah M Earl HM Poole CJ Hiller L Dunn JA, ***et al*** 2014 Association between CD8+ T-cell infiltration and breast cancer survival in 12,439 patients. Annals of Oncology 25 1536–1543. (10.1093/annonc/mdu191) 24915873

[bib3] Arteaga CL Koli KM Dugger TC Clarke R 1999 Reversal of tamoxifen resistance of human breast carcinomas in vivo by neutralizing antibodies to transforming growth factor-beta. Journal of the National Cancer Institute 91 46–53. (10.1093/jnci/91.1.46) 9890169

[bib4] Bakin AV Rinehart C Tomlinson AK Arteaga CL 2002 p38 mitogen-activated protein kinase is required for TGFbeta-mediated fibroblastic transdifferentiation and cell migration. Journal of Cell Science 115 3193–3206. 12118074 10.1242/jcs.115.15.3193

[bib5] Coffelt SB Kersten K Doornebal CW Weiden J Vrijland K Hau CS Verstegen NJM Ciampricotti M Hawinkels LJAC Jonkers J, ***et al*** 2015 IL-17-producing gammadelta T cells and neutrophils conspire to promote breast cancer metastasis. Nature 522 345–348. (10.1038/nature14282) 25822788 PMC4475637

[bib6] Colleoni M Sun Z Price KN Karlsson P Forbes JF Thurlimann B Gianni L Castiglione M Gelber RD Coates AS, ***et al*** 2016 Annual hazard rates of recurrence for breast cancer during 24 years of follow-up: results from the International Breast Cancer Study Group Trials I to V. Journal of Clinical Oncology 34 927–935. (10.1200/JCO.2015.62.3504) 26786933 PMC4933127

[bib7] de Assis S Khan G Hilakivi-Clarke L 2006 High birth weight increases mammary tumorigenesis in rats. International Journal of Cancer 119 1537–1546. (10.1002/ijc.21936) 16646052

[bib8] Dushyanthen S Beavis PA Savas P Teo ZL Zhou C Mansour M Darcy PK Loi S 2015 Relevance of tumor-infiltrating lymphocytes in breast cancer. BMC Medicine 13 202 (10.1186/s12916-015-0431-3) 26300242 PMC4547422

[bib9] Esserman LJ Moore DH Tsing PJ Chu PW Yau C Ozanne E Chung RE Tandon VJ Park JW Baehner FL, ***et al*** 2011 Biologic markers determine both the risk and the timing of recurrence in breast cancer. Breast Cancer Research and Treatment 129 607–616. (10.1007/s10549-011-1564-5) 21597921 PMC4324750

[bib10] Ewens A Mihich E Ehrke MJ 2005 Distant metastasis from subcutaneously grown E0771 medullary breast adenocarcinoma. Anticancer Research 25 3905–3915. 16312045

[bib11] Flegal KM Kruszon-Moran D Carroll MD Fryar CD Ogden CL 2016 Trends in obesity among adults in the United States, 2005 to 2014. JAMA 315 2284–2291. (10.1001/jama.2016.6458) 27272580 PMC11197437

[bib12] Gu JW Young E Busby B Covington J Johnson JW 2009 Oral administration of pyrrolidine dithiocarbamate (PDTC) inhibits VEGF expression, tumor angiogenesis, and growth of breast cancer in female mice. Cancer Biology and Therapy 8 514–521. (10.4161/cbt.8.6.7689) 19242105

[bib13] Guenard F Tchernof A Deshaies Y Cianflone K Kral JG Marceau P Vohl MC 2013 Methylation and expression of immune and inflammatory genes in the offspring of bariatric bypass surgery patients. Journal of Obesity 2013 492170 (10.1155/2013/492170) 23840945 PMC3693160

[bib14] Hard GC Iatropoulos MJ Jordan K Radi L Kaltenberg OP Imondi AR Williams GM 1993 Major difference in the hepatocarcinogenicity and DNA adduct forming ability between toremifene and tamoxifen in female Crl:CD(BR) rats. Cancer Research 53 4534–4541. 8402624

[bib15] Heldin CH Moustakas A 2016 Signaling receptors for TGF-beta family members. Cold Spring Harbor Perspectives in Biology 8 a022053 (10.1101/cshperspect.a022053) 27481709 PMC4968163

[bib16] Hilakivi-Clarke L Warri AM Bouker KB Zhang X Cook KL Jin L Zwart A Nguyen N Hu R Cruz MI, ***et al*** 2017 Effects of in utero exposure to ethinyl estradiol on tamoxifen resistance and breast cancer recurrence in a preclinical model. Journal of the National Cancer Institute 109 djw188 (10.1093/jnci/djw188) PMC6255695 27609189

[bib17] Joffroy CM Buck MB Stope MB Popp SL Pfizenmaier K Knabbe C 2010 Antiestrogens induce transforming growth factor beta-mediated immunosuppression in breast cancer. Cancer Research 70 1314–1322. (10.1158/0008-5472.CAN-09-3292) 20145137

[bib18] Johnstone CN Smith YE Cao Y Burrows AD Cross RS Ling X Redvers RP Doherty JP Eckhardt BL Natoli AL, ***et al*** 2015 Functional and molecular characterisation of EO771.LMB tumours, a new C57BL/6-mouse-derived model of spontaneously metastatic mammary cancer. Disease Models and Mechanisms 8 237–251. (10.1242/dmm.017830) 25633981 PMC4348562

[bib19] Jordan VC 1983 Laboratory studies to develop general principles for the adjuvant treatment of breast cancer with antiestrogens: problems and potential for future clinical applications. Breast Cancer Research and Treatment 3 (Supplement) S73–S86. (10.1007/BF01855131) 6423014

[bib20] Katsuno Y Lamouille S Derynck R 2013 TGF-beta signaling and epithelial-mesenchymal transition in cancer progression. Current Opinion in Oncology 25 76–84. (10.1097/CCO.0b013e32835b6371) 23197193

[bib21] Kisanga ER Gjerde J Guerrieri-Gonzaga A Pigatto F Pesci-Feltri A Robertson C Serrano D Pelosi G Decensi A Lien EA 2004 Tamoxifen and metabolite concentrations in serum and breast cancer tissue during three dose regimens in a randomized preoperative trial. Clinical Cancer Research 10 2336–2343. (10.1158/1078-0432.ccr-03-0538) 15073109

[bib22] Lee DG Burstyn I Lai AS Grundy A Friesen MC Aronson KJ Spinelli JJ 2019 Women’s occupational exposure to polycyclic aromatic hydrocarbons and risk of breast cancer. Occupational and Environmental Medicine 76 22–29. (10.1136/oemed-2018-105261) 30541747 PMC9366896

[bib23] Leibowitz KL Moore RH Ahima RS Stunkard AJ Stallings VA Berkowitz RI Chittams JL Faith MS Stettler N 2012 Maternal obesity associated with inflammation in their children. World Journal of Pediatrics 8 76–79. (10.1007/s12519-011-0292-6) 21874618

[bib24] Mao Y Qu Q Zhang Y Liu J Chen X Shen K 2014 The value of tumor infiltrating lymphocytes (TILs) for predicting response to neoadjuvant chemotherapy in breast cancer: a systematic review and meta-analysis. PLoS ONE 9 e115103 (10.1371/journal.pone.0115103) 25501357 PMC4264870

[bib25] Martin SA Jameson CH Allan SM Lawrence CB 2014 Maternal high-fat diet worsens memory deficits in the triple-transgenic (3xTgAD) mouse model of Alzheimer’s disease. PLoS ONE 9 e99226 (10.1371/journal.pone.0099226) 24918775 PMC4053375

[bib26] Mathot P Grandin M Devailly G Souaze F Cahais V Moran S Campone M Herceg Z Esteller M Juin P, ***et al*** 2017 DNA methylation signal has a major role in the response of human breast cancer cells to the microenvironment. Oncogenesis 6 e390 (10.1038/oncsis.2017.88) 29058695 PMC5668886

[bib27] Michels KB Trichopoulos D Robins JM Rosner BA Manson JE Hunter DJ Colditz GA Hankinson SE Speizer FE Willett WC 1996 Birthweight as a risk factor for breast cancer. Lancet 348 1542–1546. (10.1016/S0140-6736(96)03102-9) 8950880

[bib28] Montales MT Melnyk SB Liu SJ Simmen FA Liu YL Simmen RC 2016 Metabolic history impacts mammary tumor epithelial hierarchy and early drug response in mice. Endocrine-Related Cancer 23 677–690. (10.1530/ERC-16-0136) 27402613 PMC4997088

[bib29] Morris PG Hudis CA Giri D Morrow M Falcone DJ Zhou XK Du B Brogi E Crawford CB Kopelovich L, ***et al*** 2011 Inflammation and increased aromatase expression occur in the breast tissue of obese women with breast cancer. Cancer Prevention Research 4 1021–1029. (10.1158/1940-6207.CAPR-11-0110) 21622727 PMC3131426

[bib30] Myles IA Fontecilla NM Janelsins BM Vithayathil PJ Segre JA Datta SK 2013 Parental dietary fat intake alters offspring microbiome and immunity. Journal of Immunology 191 3200–3209. (10.4049/jimmunol.1301057) PMC3831371 23935191

[bib31] Nachat-Kappes R Pinel A Combe K Lamas B Farges MC Rossary A Goncalves-Mendes N Caldefie-Chezet F Vasson MP Basu S 2012 Effects of enriched environment on COX-2, leptin and eicosanoids in a mouse model of breast cancer. PLoS ONE 7 e51525 (10.1371/journal.pone.0051525) 23272114 PMC3521763

[bib32] Nizari S Carare RO Hawkes CA 2016 Increased Abeta pathology in aged Tg2576 mice born to mothers fed a high fat diet. Scientific Reports 6 21981 (10.1038/srep21981) 26911528 PMC4766411

[bib34] Odaka Y Nakano M Tanaka T Kaburagi T Yoshino H Sato-Mito N Sato K 2010 The influence of a high-fat dietary environment in the fetal period on postnatal metabolic and immune function. Obesity 18 1688–1694. (10.1038/oby.2009.513) 20111014

[bib35] Ogden CL Carroll MD Fryar CD Flegal KM 2015 Prevalence of obesity among adults and youth: United States, 2011–2014. NCHS Data Brief 219 1–8. (available at: https://www.cdc.gov/nchs/data/databriefs/db219.pdf) 26633046

[bib33] O’Reilly JR Reynolds RM 2013 The risk of maternal obesity to the long-term health of the offspring. Clinical Endocrinology 78 9–16. (10.1111/cen.12055) 23009645

[bib36] Pan H Gray R Braybrooke J Davies C Taylor C McGale P Peto R Pritchard KI Bergh J Dowsett M, ***et al*** 2017 20-year risks of breast-cancer recurrence after stopping endocrine therapy at 5 years. New England Journal of Medicine 377 1836–1846. (10.1056/NEJMoa1701830) 29117498 PMC5734609

[bib37] Park EJ Lee JH Yu GY He G Ali SR Holzer RG Osterreicher CH Takahashi H Karin M 2010 Dietary and genetic obesity promote liver inflammation and tumorigenesis by enhancing IL-6 and TNF expression. Cell 140 197–208. (10.1016/j.cell.2009.12.052) 20141834 PMC2836922

[bib38] Pink JJ Jordan VC 1996 Models of estrogen receptor regulation by estrogens and antiestrogens in breast cancer cell lines. Cancer Research 56 2321–2330. 8625307

[bib39] Russo J 2015 Significance of rat mammary tumors for human risk assessment. Toxicologic Pathology 43 145–170. (10.1177/0192623314532036) 25714400 PMC4358771

[bib40] Sengupta P 2013 The laboratory rat: relating its age with human’s. International Journal of Preventive Medicine 4 624–630. 23930179 PMC3733029

[bib41] Shoelson SE Herrero L Naaz A 2007 Obesity, inflammation, and insulin resistance. Gastroenterology 132 2169–2180. (10.1053/j.gastro.2007.03.059) 17498510

[bib42] Silva Idos S De Stavola B McCormack V & Collaborative Group on Pre-Natal Risk Factors and Subsequent Risk of Breast Cancer 2008 Birth size and breast cancer risk: re-analysis of individual participant data from 32 studies. PLoS Medicine 5 e193 (10.1371/journal.pmed.0050193) 18828667 PMC2553821

[bib43] Singh S Chakrabarti R 2019 Consequences of EMT-driven changes in the immune microenvironment of breast cancer and therapeutic response of cancer cells. Journal of Clinical Medicine 8 642 (10.3390/jcm8050642) PMC6572359 31075939

[bib44] Sobral-Leite M Salomon I Opdam M Kruger DT Beelen KJ Van der Noort V van Vlierberghe RLP Blok EJ Giardiello D Sanders J, ***et al*** 2019 Cancer-immune interactions in ER-positive breast cancers: PI3K pathway alterations and tumor-infiltrating lymphocytes. Breast Cancer Research 21 90 (10.1186/s13058-019-1176-2) 31391067 PMC6686400

[bib45] Soundararajan R Fradette JJ Konen JM Moulder S Zhang X Gibbons DL Varadarajan N Wistuba II Tripathy D Bernatchez C, ***et al*** 2019 Targeting the interplay between epithelial-to-mesenchymal-transition and the immune system for effective immunotherapy. Cancers 11 E714 (10.3390/cancers11050714) 31137625 PMC6562947

[bib46] Sovio U Jones R Dos Santos Silva I Koupil I 2013 Birth size and survival in breast cancer patients from the Uppsala Birth Cohort Study. Cancer Causes and Control 24 1643–1651. (10.1007/s10552-013-0238-5) 23722348

[bib47] Sun X Casbas-Hernandez P Bigelow C Makowski L Joseph JD Smith SS Troester MA 2011 Normal breast tissue of obese women is enriched for macrophage markers and macrophage-associated gene expression. Breast Cancer Research and Treatment 131 1003–1012. 22002519 10.1007/s10549-011-1789-3 PMC3640411

[bib48] Suzuki H Onishi H Wada J Yamasaki A Tanaka H Nakano K Morisaki T Katano M 2010 VEGFR2 is selectively expressed by FOXP3high CD4+ Treg. European Journal of Immunology 40 197–203. (10.1002/eji.200939887) 19902430

[bib49] Terry S Savagner P Ortiz-Cuaran S Mahjoubi L Saintigny P Thiery JP Chouaib S 2017 New insights into the role of EMT in tumor immune escape. Molecular Oncology 11 824–846. (10.1002/1878-0261.12093) 28614624 PMC5496499

[bib50] White AJ Bradshaw PT Herring AH Teitelbaum SL Beyea J Stellman SD Steck SE Mordukhovich I Eng SM Engel LS, ***et al*** 2016 Exposure to multiple sources of polycyclic aromatic hydrocarbons and breast cancer incidence. Environment International 89–90 185–192. (10.1016/j.envint.2016.02.009) PMC4818720 26878284

[bib51] Wilson RM Marshall NE Jeske DR Purnell JQ Thornburg K Messaoudi I 2015 Maternal obesity alters immune cell frequencies and responses in umbilical cord blood samples. Pediatric Allergy and Immunology 26 344–351. (10.1111/pai.12387) 25858482 PMC9271931

[bib52] Yu Z Han S Zhu J Sun X Ji C Guo X 2013 Pre-pregnancy body mass index in relation to infant birth weight and offspring overweight/obesity: a systematic review and meta-analysis. PLoS ONE 8 e61627 (10.1371/journal.pone.0061627) 23613888 PMC3628788

[bib53] Zhang S 2018 The role of transforming growth factor beta in T helper 17 differentiation. Immunology 155 24–35. (10.1111/imm.12938) 29682722 PMC6099164

[bib54] Zhang X Cook KL Warri A Cruz IM Rosim M Riskin J Helferich W Doerge D Clarke R Hilakivi-Clarke L 2017 Lifetime genistein intake increases the response of mammary tumors to tamoxifen in rats. Clinical Cancer Research 23 814–824. (10.1158/1078-0432.CCR-16-1735) 28148690 PMC5654585

