# Supplementary figures and images for: Maternal obesity increases offspring’s mammary cancer recurrence and impairs tumor immune response
Source: Endocr Relat Cancer. 2020 Jun 22;27(9):469–82. doi: 10.1530/ERC-20-0065 (PMC7424355; doi:10.1530/ERC-20-0065)

A

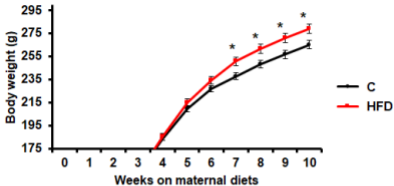

B

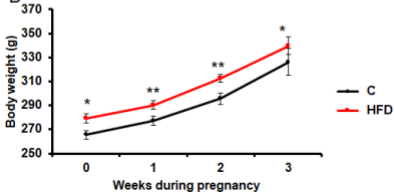

Supplement: Supplementary Figure 1. Weight gain in obesity-inducing high fat diet (HFD) fed dams before and during pregnancy. (A) Weight gain of dam rats before pregnancy. Seven weeks of HFD feeding led to a significantly higher weight gain, compared with control [C] diet. Rats were kept on these diets for thre [file supplementary_figure_1.pdf]

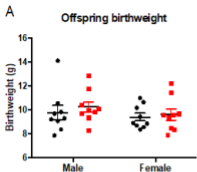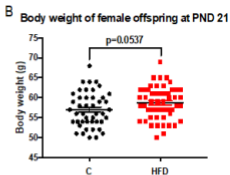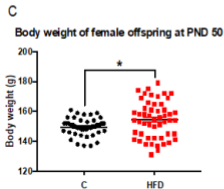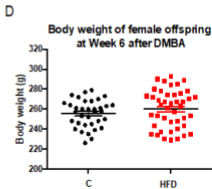

Supplement: Supplementary Figure 2. Body weight of offspring of dams fed obesity-inducing high fat diet (HFD) or control [C] diet. (A) Birthweight of male and female offspring of control and HFD fed dams. (B) Body weight of female offspring at postnatal day (PND) 21 and (C) PND 50. (D) Body weight of female off [file supplementary_figure_2.pdf]

**A**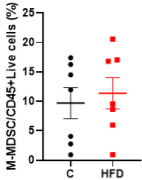**B**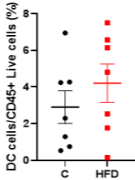**C**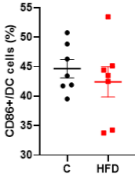

Supplement: Supplementary Figure 4. Effect of maternal obesity-inducing high fat diet (HFD) on immune markers in E0771 mammary tumors of mouse offspring. Frequency of (A) monocytic-Myeloid-derived suppressor cells (M-MDSC: CD45+CD3-CD11b+CD11c-Ly6C+Ly6G--F4/80-) (B) Dendritic cells (DC: CD45+CD3-CD11b+CD11c+F4/ [file supplementary_figure_4.pdf]
