# Supplementary material for: Maternal obesity increases offspring’s mammary cancer recurrence and impairs tumor immune response
Source: Endocr Relat Cancer. 2020 Jun 22;27(9):469–82. doi: 10.1530/ERC-20-0065 (PMC7424355; doi:10.1530/ERC-20-0065)
Supplement: Supplementary Figure 3. Effect of maternal obesity-inducing high fat diet (HFD) on MHCI in the rat mammary tumors of offspring. (A) Gene expression of RT1.A1 and (B) RT1.EC2 in rat mammary tumors from control [C] (black circles) and HFD (red squares) offspring before TAM treatment, and in TAM-treate [file supplementary_figure_3.pdf]

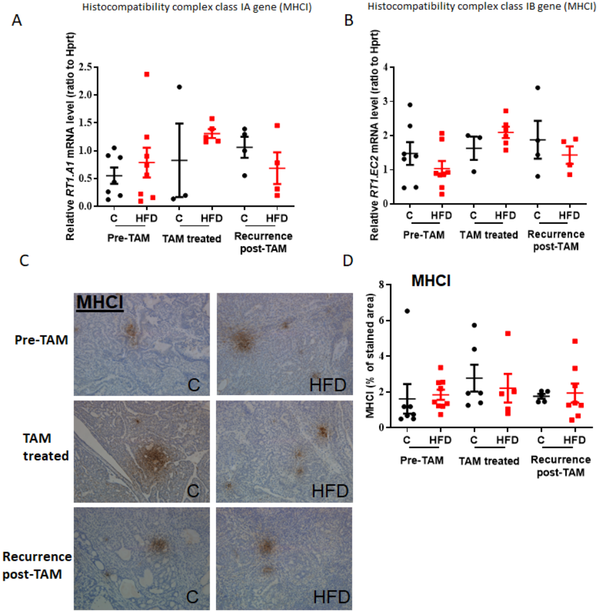

A
Histocompatibility complex class IA gene (MHCI)
Relative RT1.A1 mRNA level (ratio to Hprt)
0.0
0.5
1.0
1.5
2.0
2.5
C
HFD
C
HFD
C
HFD
Pre-TAM
TAM treated
Recurrence post-TAM
B
Histocompatibility complex class IB gene (MHCI)
Relative RT1.EC2 mRNA level (ratio to Hprt)
0
1
2
3
4
C
HFD
C
HFD
C
HFD
Pre-TAM
TAM treated
Recurrence post-TAM
C
MHCI
Pre-TAM
TAM treated
Recurrence post-TAM
C
HFD
C
HFD
C
HFD
D
MHCI
MHCI (% of stained area)
0
2
4
6
8
C
HFD
C
HFD
C
HFD
Pre-TAM
TAM treated
Recurrence post-TAM
