# Supplementary material for: Maternal obesity increases offspring’s mammary cancer recurrence and impairs tumor immune response
Source: Endocr Relat Cancer. 2020 Jun 22;27(9):469–82. doi: 10.1530/ERC-20-0065 (PMC7424355; doi:10.1530/ERC-20-0065)
Supplement: Supplementary Figure 5. Effect of maternal obesity-inducing high fat diet (HFD) on hormone receptor levels, apoptosis and cell proliferation in the mammary tumors of rat offspring. (A) Maternal HFD increased ERα protein level in the TAM-treated tumors, when compared with TAM-treated tumors in contro [file supplementary_figure_5.pdf]

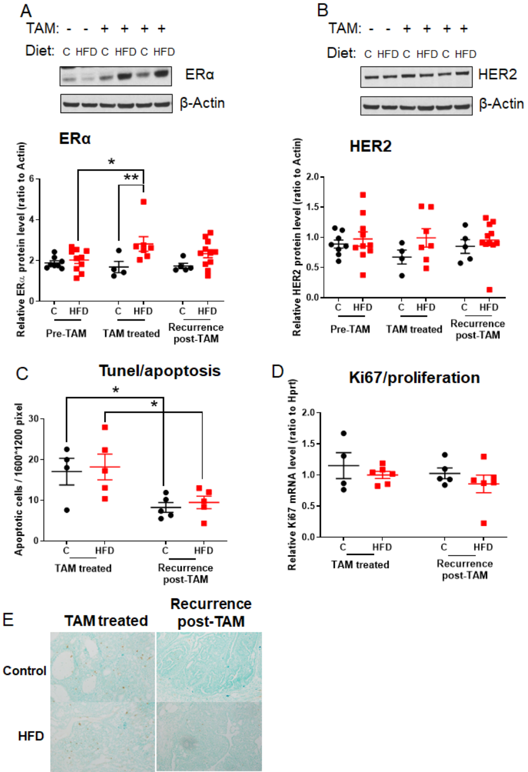

A
TAM: - - + + + +
Diet: C HFD C HFD C HFD
ERα
β-Actin
B
TAM: - - + + + +
Diet: C HFD C HFD C HFD
HER2
β-Actin
ERα
Relative ERα protein level (ratio to Actin)
*
**
0
2
4
6
C HFD C HFD C HFD
Pre-TAM
TAM treated
Recurrence post-TAM
HER2
Relative HER2 protein level (ratio to Actin)
0.0
0.5
1.0
1.5
2.0
C HFD C HFD C HFD
Pre-TAM
TAM treated
Recurrence post-TAM
C
Tunel/apoptosis
Apoptotic cells / 1600*1200 pixel
*
*
0
10
20
30
C HFD C HFD
TAM treated
Recurrence post-TAM
D
Ki67/proliferation
Relative Ki67 mRNA level (ratio to Hprt)
0.0
0.5
1.0
1.5
2.0
C HFD C HFD
TAM treated
Recurrence post-TAM
E
TAM treated
Recurrence post-TAM
Control
HFD
