# Supplementary material for: Maternal obesity increases offspring’s mammary cancer recurrence and impairs tumor immune response
Source: Endocr Relat Cancer. 2020 Jun 22;27(9):469–82. doi: 10.1530/ERC-20-0065 (PMC7424355; doi:10.1530/ERC-20-0065)
Supplement: Supplementary Table 1. Ingredients of control and high fat diets for rats [file supplementary_table_1.pdf]

Supplementary Table 1. Ingredients of control and high fat diets for rats

Ingredient	Control g/Kg	HFD
Casein	207.0	207.0
L-Cystine	3.0	3.0
Sucrose	100.0	230.0
Maltodextrin	130.0	100.0
Corn Starch	412.49	80.65
Cellulose	50.0	50.0
Vegetable Shortening, Hydrogenated (Crisco)	25	215.0
Corn Oil	25	50.0
Mineral Mix, AIN-93G-MX (94046)	35.0	46.0
Calcium Phosphate, monobasic, monohydrate	0	1.5
Vitamin Mix, AIN-93-VX (94047)	10.0	13.5
Choline Bitartrate	2.5	3.3
TBHQ, antioxidant	0.01	0.05
	% kcal from	
Protein	20.1	15.2
Carbohydrate	67.1	34.9
Fat	12.8	49.9
Kcal/g	3.7	4.8
