# Supplementary material for: Maternal obesity increases offspring’s mammary cancer recurrence and impairs tumor immune response
Source: Endocr Relat Cancer. 2020 Jun 22;27(9):469–82. doi: 10.1530/ERC-20-0065 (PMC7424355; doi:10.1530/ERC-20-0065)
Supplement: Supplementary Table 2. Ingredients of control and high fat diets for mice [file supplementary_table_2.pdf]

**Supplementary Table 2**. Ingredients of control and high fat diets for mice

| Ingredient | Control<br>g/Kg | HFD |
|---|---|---|
| Casein | 210.0 | 265.0 |
| L-Cystine | 3.0 | 4.0 |
| Sucrose | 90.0 | 90 |
| Maltodextrin | 100.0 | 160.0 |
| Corn Starch | 465.0 | 0 |
| Cellulose | 37.15 | 65.5 |
| Lard | 20 | 310.0 |
| Soybean Oil | 20 | 30.0 |
| Mineral Mix, AIN-93G-MX (94046) | 35.0 | 48.0 |
| Calcium Phosphate, dibasic | 2.0 | 3.4 |
| Vitamin Mix, AIN-93-VX (94047) | 15.0 | 21.0 |
| Choline Bitartrate | 2.75 | 3.0 |
| | % kcal from | |
| Protein | 20.5 | 18.3 |
| Carbohydrate | 69.1 | 21.4 |
| Fat | 10.5 | 60.3 |
| Kcal/g | 3.6 | 5.1 |
