# Supplementary material for: Maternal obesity increases offspring’s mammary cancer recurrence and impairs tumor immune response
Source: Endocr Relat Cancer. 2020 Jun 22;27(9):469–82. doi: 10.1530/ERC-20-0065 (PMC7424355; doi:10.1530/ERC-20-0065)
Supplement: Supplementary Table 3. Primers used in quantitative real-time PCR [file supplementary_table_3.pdf]

Supplementary Table 3. Primers used in quantitative real-time PCR

Rat: Gene	Sequence
*Cdh1*_ forward	5’- AACGCTCCCATCTTCAACC-3’
*Cdh1*_reverse	5’-ATCAGGATCATTGACTACGGTG-3’
Hprt_forward	*5’- CCCCAAAATGGTTAAGGTTGC -3’*
Hprt_reverse	*5’- AACAAAGTCTGGCCTGTATCC -3’*
*Il-6*_forward	5’- TTGCCTTCTTGGGACTGATG -3’
*Il-6*_reverse	5’- GTGGTATCCTCTGTGAAGTCTC -3’
*Il-17c*_forward	5’- GAAGCTGACACCCACGAG -3’
Il-17c_reverse	5’- ACCCCGACACAAGCATTC -3’
*Il-17f*_forward	5’- CAAAACCAGGGCATTTCTGTC -3’
*Il-17f*_reverse	5’- GTTGATACAGCCTGAGTGTCTG -3’
*Ki67*_forward	5’- ATTCAGGCCCTGCGAAGCCG -3’
*Ki67*_reverse	5’- GCGTTGAAGGTAGGTGCCCCA -3’
Rt1.A1_forward	*5’-GAGCCACTTTCCCAGAGATG-3’*
Rt1.A1_ reverse	*5’-TTCCTCACAACAGTCACCAC-3’*
Rt1.Ec2_ forward	*5’- CATCCACCGACTCCAACTTAC-3’*
Rt1.Ec2_reverse	*5’- TCCTCACAACAACCACCATAG-3’*
Rt1.Bb_ forward	*5’- CTGCAGACACAACTACGAGG-3’*
Rt1.Bb_ reverse	*5’- GGCTGGGTAGAAATCTGTCAC-3’*
Rt1.Da_ forward	*5’- AATGTCCTGTGTGTTCTCGG-3’*
Rt1.Da_reverse	*5’- CTCTCACGGAAGCCATCAC-3’*

*Tgfβ1*_forward	5’- CCTGAGTGGCTGTCTTTTGA -3’
*Tgfβ1*_reverse	5’- CGTGGAGTACATTATCTTTGCTG -3’

Mouse: Gene	Sequence
Cdh1_forward	*5’- AGACGCTGAGCATGTGAA -3’*
Cdh1_reverse	*5’-TGTTTCGAGGTTCTGGGATG -3’*
Il-*6*_forward	5’- GTTCCTCTCTGCAAGAGACTTCC-3’
Il-*6*_reverse	5’-ACAGGTCTGTTGGGAGTGGTATC -3’
*Il-17c*_forward	5’-GGAGATATCGCATCGACACAGA -3’
Il-17c_reverse	5’-GCATCCACGACACAAGCATT -3’
*Il-17f*_forward	5’-GTGAAACAGCCATGGTCAAGTC -3’
*Il-17f*_reverse	5’-GGTTCTTCCGAGCTGCTACC -3’
Tbp_forward	*5’-AGGATGCTCTAGGGAAGATCTGAG -3’*
Tbp_reverse	*5’-GAGCATAAGGTGGAAGGCTGTT -3’*
